# Clinical epidemiology and a novel predicting nomogram of central line associated bloodstream infection in burn patients

**DOI:** 10.1017/S0950268823000766

**Published:** 2023-05-23

**Authors:** Yangping Wang, Qimeng Li, Qin Shu, Menglong Liu, Ning Li, Wen Sui, Zhiqiang Yuan, Gaoxing Luo, Haisheng Li

**Affiliations:** 1 Institute of Burn Research, State Key Laboratory of Trauma, Burns and Combined Injury, Southwest Hospital, Third Military Medical University (Army Medical University), Chongqing China; 2 Laboratory of Trauma Care, School of Nursing, Third Military Medical University (Army Medical University), Chongqing China; 3 Center for Joint Surgery, Southwest Hospital, Third Military Medical University (Army Medical University), Chongqing China

**Keywords:** Burns, central line–associated bloodstream infection, epidemiology, nomogram, prediction

## Abstract

Burn patients are at high risk of central line–associated bloodstream infection (CLABSI). However, the diagnosis of such infections is complex, resource-intensive, and often delayed. This study aimed to investigate the epidemiology of CLABSI and develop a prediction model for the infection in burn patients. The study analysed the infection profiles, clinical epidemiology, and central venous catheter (CVC) management of patients in a large burn centre in China from January 2018 to December 2021. In total, 222 burn patients with a cumulative 630 CVCs and 5,431 line-days were included. The CLABSI rate was 23.02 CVCs per 1000 line-days. The three most common bacterial species were *Acinetobacter baumannii, Staphylococcus aureus*, and *Pseudomonas aeruginosa*; 76.09% of isolates were multidrug resistant. Compared with a non-CLABSI cohort, CLABSI patients were significantly older, with more severe burns, more CVC insertion times, and longer total line-days, as well as higher mortality. Regression analysis found longer line-days, more catheterisation times, and higher burn wounds index to be independent risk factors for CLABSI. A novel nomogram based on three risk factors was constructed with an area under the receiver operating characteristic curve (AUROC) value of 0.84 (95% CI: 0.782–0.898) with a mean absolute error of calibration curve of 0.023. The nomogram showed excellent predictive ability and clinical applicability, and provided a simple, practical, and quantitative strategy to predict CLABSI in burn patients.

## Introduction

Burns represent a common type of trauma worldwide [1]. The World Health Organization estimates the occurrence of about 11 million burns and the death of 300,000 burn patients annually worldwide, resulting in the loss of about 18 million disability-adjusted life years [2, 3]. Approximately 90% of burn injuries were documented in low- and middle-income regions, including China [2], and remain a major public health concern. Central venous catheters (CVCs) are indispensable for burn treatment as they provide stable vascular access for fluid resuscitation, safe infusion of antibacterial and vasoactive drugs [4], parenteral nutritional support therapy [5], continuous hemodialysis therapy [6], and continuous hemodynamic monitoring [7].

Catheter-related infection (CRI) and thrombosis are the two major, sometimes lethal, complications of CVCs, with central line–associated bloodstream infection (CLABSI) being the most serious presentation, and characterised by a significantly higher incidence – two- to three-fold – in burn patients (15.5–29.1 per 1,000 catheter days) than in other patients (4.80–8.64 per 1,000 catheter days) [8-11].

CLABSI not only prolongs the patient’s hospital stay and increases the medical burden [8] but also may affect clinical outcomes and increase morbidity and mortality [9, 10]. It has become one of the key indicators of nosocomial infections worldwide, with serious complications ranging from sepsis syndromes, endocarditis, and haematogenous transmission, which leads to additional healthcare, estimated in one study to be increased over two-fold [11]. More importantly, CLABSI is an independent risk factor for death [12], and as such its prevention and treatment have become priorities in hospital infection control.

In essence, CLABSI is a diagnosis of exclusion, and requires other infections (wound, respiratory, and urinary tracts) to be ruled out first. Consequently, its diagnosis may be delayed and may impact negatively on appropriate treatment [13]. However, as most current studies focus on the screening of risk factors for CLABSI, a quantitative method to predict the infection remains lacking in clinical practice. A nomogram is a common presentation of a prediction model, which can quantitatively integrate multiple risk factors with different ratios [14]. In practice, a total risk score can be calculated by the actual value of several variables and give the risk probability of a specific infection in a patient or population, with an obvious benefit for their prevention and treatment.

In this study, we analysed retrospectively the clinical features, infection profiles, and related risk factors of CLABSI in burn patients through the construction of a novel nomogram with the aim to provide a simple, practical, and quantitative support system for the timely diagnosis, and treatment of affected patients.

## Methods

### Study design

A retrospective study was conducted at the Institute of Burn Research, Southwest Hospital, Third Military Medical University, between January 2018 and December 2021. This is one of the largest burn centres in the world and has 126 inpatient beds (including 18 beds in a burn intensive care unit), and annually admits approximately 1,300 patients from southwest China. The study was approved by the ethical committee of the Southwest Hospital (No. KY202245). In accordance with national legislation and institutional requirements, written informed consent from the participants’ legal guardian / next of kin was not required to participate in this study.

### Data collection

Patients with CVCs were primarily identified through a search of the hospital’s burn database. Only burn patients were included; others with CVCs inserted before admission, or no documented bacterial cultures or incomplete data were excluded, along with patients admitted later than 1 month after burn injury. In total, 222 patients with 630 CVC events were included. The following data were extracted from medical records: demographic data (gender, age, admission date, injury date, BMI), clinical features (burn aetiology, area and location, inhalation injury, infection, transfusion, in-bed days, operation numbers), CVC information (insertion date, puncturing times, specific veins and sides, tip location, extubation date, and others), as well as CLABSI details (pathogen name, detecting time, drug resistance), and outcomes (death, length of hospital stay).

### CVCs insertion and management

The application of CVC was determined by doctors, mainly based on the following considerations: fluid resuscitation, long-term parenteral nutrient supply, infusion of antimicrobials, vasoactive and other stimulus agents, repeated blood tests, and continuous blood purification treatment. All CVCs were inserted by an experienced team according to practice guidelines [15]. All operators wore masks, caps, sterile gloves, and surgical gowns. Hand disinfection with 70% ethanol by volume or other hand disinfection solutions was mandatory before and after catheter placement, replacement, viewing, adjustment, or dressing change. For placement via deep burn wounds, 20 g/L tincture of iodine was used, and via superficial burn wounds, chlorhexidine or other iodine-containing disinfectants were used.

Ultrasound was used to guide the catheterisation of CVCs when venipuncture catheterisation was difficult, and if the catheter was inserted in a burn wound, gauze with povidone-iodine was applied to the catheter exit site. Dressings were changed, and the length of the outer catheter was measured daily. Dressings were replaced if they appeared damp, loose, or visibly polluted. Catheter puncture points and signs of systemic infection were observed daily, and if local inflammation at the puncture site or vascular CRI was suspected, a comprehensive evaluation was made by the medical team to determine whether extubation was necessary. If CLABSI was suspected, peripheral blood samples were collected for microbiological culture before, or immediately after, catheter removal.

### Microbiological identification and drug sensitivity test

Microbiological culture was performed by semi-quantitative or quantitative methods. The proximal tip of the extracted catheter (about 5 cm long) was rolled several times over a Columbia blood agar plate medium (Difco, Franklin Lakes, NJ, USA) in a ‘Z’ pattern, and following incubation, microbial growths were identified by standard microbiological procedures. A panel of culture agar media (blood, chocolate, etc) was used for the semi-quantitation of microbial loads in clinical samples. All media were incubated for 18–24 h in atmospheres appropriate for the species sought. Isolates were identified to species level and antimicrobial susceptibility determined using the VITEK-2 compact system analysis (BioMerieux, Saint-Vulbas, France), with reference to the Clinical and Laboratory Standards Institute for MIC breakpoint determination. Breakpoint concentrations of cefoxitin of ≤4 and ≥ 8 mg/L were used to differentiate between methicillin-susceptible and methicillin-resistant *Staphylococcus aureus* isolates. Multiple drug-resistant (MDR) and extensively drug-resistant (XDR) strains were defined as previously described [16]. Carbapenem-resistant *Acinetobacter baumannii* (CRAB), carbapenem-resistant *Pseudomonas aeruginosa* (CRPA), and carbapenem-resistant *Klebsiella pneumoniae* (CRKP) were defined by resistance to imipenem or meropenem.

### Definition of CLABSI

CLABSI was defined as the presence of a positive blood culture in a patient with an indwelling CVC, or within 48 hours after its removal [17]. The infection was diagnosed based on the following criteria: (1) the isolation of a recognised pathogen cultured from one or more blood samples; (2) a fever (>38 °C), chills, hypotension, or other signs and symptoms, as well as positive laboratory results not related to an infection at another site; and (3) isolation of the same microbial species with similar antimicrobial susceptibility profile from the blood and the catheter tip. CLABSI rates were reported as events per CVCs/1,000 line-days. For patients with multiple catheterisations, only the first positive result was diagnosed as CLABSI if consecutive positive cultures with the same microorganism occurred.

### Nomogram construction and statistical analysis

Data analysis was performed using GraphPad Prism 6 (USA, GraphPad Software Inc.) and SPSS 22.0 (USA, IBM analytics). The *t-*test was used to compare quantitative variables with nominal distribution, and the Mann–Whitney U test was conducted to compare two categorical variables. Chi-squared test was applied to assess significant associations between two categorical variables (frequency and percentage). Multicollinearity among the included variables was analysed using collinearity diagnostics prior to regression analysis. Univariate and multivariate logistic regression (forward LR method, entry: *p* = 0.05; removal = (0.10)) was used to screen for factors contributing to CLABSI. Kaplan–Meier methods were used to perform survival analysis, and Cox regression models to screen out risk factors of death. Details regarding the variable assignments are shown in Table S1.

Least-absolute shrinkage and selection operator (LASSO) regression analysis, nomogram construction, and evaluation were performed in R Studio 2022.02.0 software (Prairie Trillium Release) using ‘*glmnet*’, ‘*rms*’, ‘*pROC*’, and ‘*ggDCA*’ packages. LASSO regression analysis was performed to determine associations between risk factors and CLABSI. All candidate features were entered into the analysis, and the assumption of proportional hazards was confirmed. The optimal feature combination was selected based on the LASSO regression analysis. The nomogram was constructed on the optimal feature, and evaluated by the area under the receiver operating characteristic curve (AUROC), calibration curves, and decision curve analysis. P values < 0.05 were considered statistically significant.

## Results

**Figure 1.**
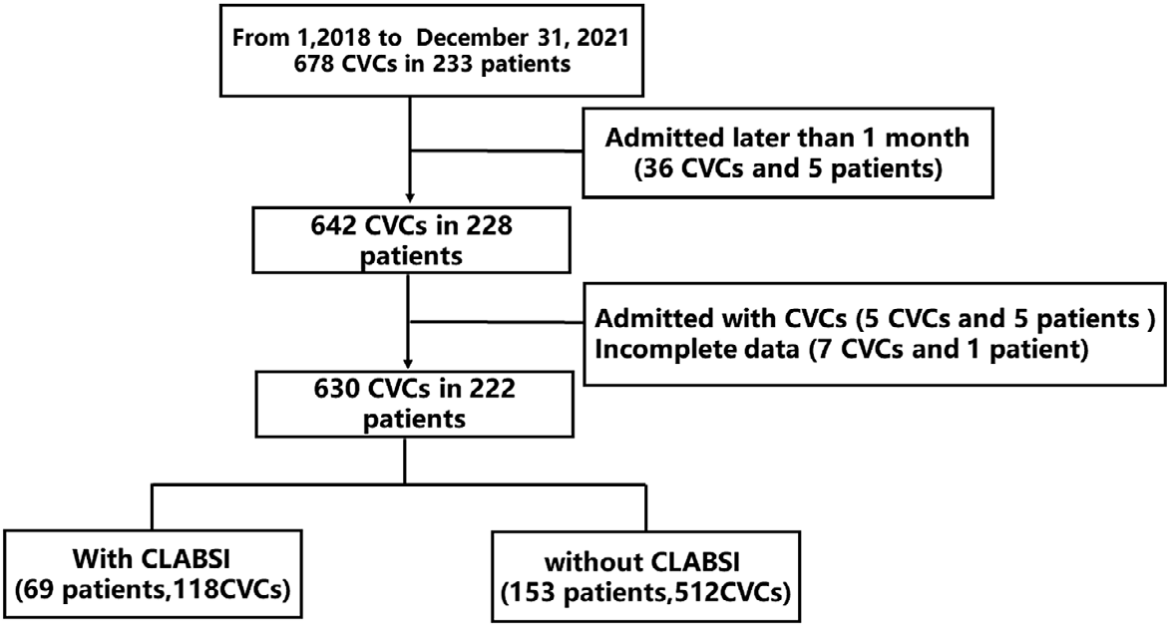
Flow chart of patient selection.

**Figure 2.**
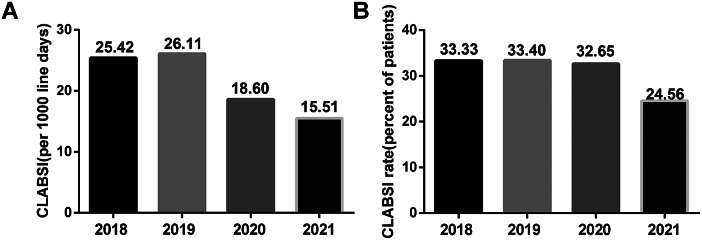
The annual incidence of CLABSI in burn patients. (a) The annual incidence of CVCs with CLABSI per 1,000-day line-days. (b) The annual percentage of burn patients with CLABSI in total burn patients.

A total of 233 patients with 678 CVCs were initially included, which was then reduced after step-by-step selection to 222 patients with 630 CVCs (Figure 1). Among 630 CVCs, a total of 118 CLABSI cases were identified, with a 1,000 line-day infection rate of 23.02. The annual incidence of CVCs with CLABSI showed an overall downward trend from 2018 to 2021 (25.42, 26.11, 18.60, and 15.51, respectively) (Figure 2a). Among the 222 patients’ cohort, 69 developed CLABSI, with a rate of 31.08%. Overall, the annual percentage of burn patients with CLABSI also gradually decreased, particularly in 2021 (Figure 2b).

### Antimicrobial resistance of bacterial isolates

As shown in supplementary Table S2, 163 bacterial isolates were identified, and over three-quarters were Gram-negative species. The three most common were *A. baumannii*, *P. aeruginosa*, and *S. aureus* accounting for 39.26%, 18.40%, and 12.88%, respectively.

The rate of antibacterial resistance was relatively high, with 76.09% of isolates classed as MDR, of which 18.48% were XDR. All isolates of *A. baumannii* were MDR (42.2% XDR), and 75.0% of *P. aeruginosa* were also MDR (18.7% XDR). Likewise, all *S. aureus* isolates were MRSA. Carbapenem resistance was universal in *A. baumannii,* and also evident in 62.5% of *P. aeruginosa.* All *A. baumannii* were susceptible to polymyxin B, tigecycline, and minocycline, and exhibited variable resistance to amikacin, gentamicin, levofloxacin, and tobramycin (42.4–87.9%) (Table 1). Likewise, *P. aeruginosa* were uniformly susceptible to polymyxin B, with varying resistance rates to meropenem and other antimicrobials, but resistant to cotrimoxazole; 62.5% were resistant to meropenem and moderately so to other antibiotics (31.3%–60.0%) (Table 1). *S.aureus* isolates were uniformly susceptible to vancomycin, teicoplanin, linezolid, and tigecycline, but highly resistant to other antibiotics (Table 1).

**Table 1.** The distribution of drug resistance of pathogens in CLABSI

*A. baumannii*		*P. aeruginosa*		*S. aureus*	
Antibiotics	Resistance rate (%)	Antibiotics	Resistance rate (%)	Antibiotics	Resistance rate (%)
Amikacin	87.9	Amikacin	31.3	Ciprofloxacin	100
Aztreonam	100	Aztreonam	31.3	Erythromycin	100
Cefepime	100	Cefepime	50	Gentamicin	94.4
Cefoperazone/sulbactam	100	Cefoperazone/sulbactam	50	Levofloxacin	100
Ceftazidime	100	Ceftazidime	50	Linezolid	0
Ciprofloxacin	97	Ciprofloxacin	43.8	Moxifloxacin	100
Gentamicin	75.8	Gentamicin	43.8	Oxacillin	94.4
Imipenem	100	Imipenem	68.8	Penicillin	100
Levofloxacin	42.4	Levofloxacin	43.8	Quinupristin	0
Meropenem	100	Meropenem	62.5	Rifampicin	100
Minocycline	0	Piperacillin	50	Teicoplanin	0
Piperacillin	100	Polymyxin B	0	Tetracycline	100
Polymyxin B	0	Tobramycin	60	TMPco	94.4
Sulfamethoxazole-trimethoprim	93.9	Sulfamethoxazole-trimethoprim	100	Vancomycin	0
Tigecycline	0				
Tobramycin	78.8				

### Clinical features of patients with and without CLASBI

The demographics and clinical features of the included 222 patients with and without CLABSI are shown in Table 2. CLABSI patients comprised significantly more males (5.3:1 vs. 3.0:1, *p* = 0.002), were of older age (42.04 ± 14.37 vs. 41.94 ± 18.89, *p* = 0.021), and presented with more flame burns (69.57% vs. 67.32%, *p* = 0.038). Burn severity was significantly higher in the CLABSI group, including total burn area (68.87 ± 22.31 vs. 46.26 ± 20.72, *p* < 0.001), full-thickness burn area (41.10 ± 23.26 vs. 20.99 ± 16.95, *p* = 0.002), revised Baux score (122.25 ± 33.01 vs. 96.23 ± 32.14, *p* < 0.001), burn index (57.69 ± 21.34 vs. 36.02 ± 17.94, *p* < 0.001), and inhalation injury (66.67% vs. 47.06%, *p* < 0.001). Notably, CLABSI patients had significantly more line insertion times (4.74 ± 3.20 vs. 1.94 ± 1.50, *p* < 0.001), longer total line-days (43.65 ± 31.84 vs. 25.13 + 15.90, *p* < 0.001), and the longest duration (13.80 ± 7.15 vs. 11.32 + 6.73, *p* < 0.001) than the non-CLABSI group. Unsurprisingly, the mortality rate (13.04% vs. 5.23%, *p* < 0.001), in-bed days (53.00 ± 31.75 vs. 28.32 ± 21.31, *p* = 0.003), and length of hospital stay (94.06 ± 52.53 vs. 63.05 ± 39.36, *p* < 0.001) were also higher in the CLABSI group.

**Table 2.** Clinical features of burn patients with and without CLABSI

Characteristics	Total, *n* = 222	CLABSI, *n* = 69	Non-CLABSI, *n* = 153	*p-*value
Age (mean ± SD)	41.97 ± 17.58	42.04 ± 14.37	41.94 ± 18.89	0.968
Sex				0.002
Male, *n* (%)	173(77.93%)	58(84.06%)	115(75.16%)	
Female, *n* (%)	49(22.07%)	11(15.94%)	38(24.83%)	
Male: female	3.5:1	5.3:1	3.0:1	
BMI (mean ± SD)	23.03 ± 4.95	23.34 ± 3.98	22.33 ± 6.42	0.456
Burn etiology, *n* (%)				0.938
Flame burns	151(68.02%)	48(69.57%)	103(67.32%)	
Scalds	22(9.91%)	4(5.80%)	18(11.76%)	
Electric burns	29(13.06%)	10(14.49)	19(12.42%)	
Explosion	15(6.76%)	6(8.70)	9(5.88%)	
Chemical burns	5(2.25%)	1(1.45%)	4(2.61%)	
Burn severity				
Total burn area (mean ± SD)	53.31 ± 23.63	68.87 ± 22.31	46.26 ± 20.72	<0.001
Burn index (mean ± SD)	47.76 ± 21.50	57.69 ± 21.34	36.02 ± 17.94	<0.001
Baux score (mean ± SD)	104.32 ± 34.52	122.25 ± 33.01	96.23 ± 32.14	<0.001
Full-thickness burn area (mean ± SD)	27.17 ± 21.20	41.10 ± 23.26	20.99 ± 16.95	<0.001
Inhalation injury (*n*, %)	118(53.15%)	46(66.67%)	72(47.06%)	<0.001
CVC details				
Insertion times (mean ± SD)	2.81 ± 2.52	4.74 ± 3.20	1.94 ± 1.50	<0.001
Longest line duration (mean ± SD)	11.34 ± 7.04	13.80 ± 7.15	11.32 + 6.73	0.001
Total line-days (mean ± SD)	25.10 ± 25.33	43.65 ± 31.84	25.13 + 15.90	<0.001
0–7 (*n*, %)	45(20.27%)	3(4.35%)	42(27.45%)	
8–14 (*n*, %)	55(24.77%)	5(7.25%)	50(32.68%)	
15–21 (*n*, %)	36(16.21%)	11(15.94%)	25(16.34%)	
>21 (*n*, %)	86(38.74%)	50(72.46%)	36(23.53%)	
Catheterisation times (Median, IQR)	2(1–3.75)	1(1–2)	4(2–7)	<0.001
1	89(40.09%)	9(13.04%)	80(52.29%)	<0.001
2	53(23.87%)	13(18.84%)	40(26.14%)	
3	24(10.81%)	7(10.14%)	17(11.11%)	
>3	56(25.23%)	40(57.97%)	16(10.46%)	
History, *n* (%)				
Hypertension	18(8.11%)	3(4.35%)	15(9.80%)	0.004
Hyperlipoidemia	8(3.60%)	1(1.45%)	7(4.58%)	0.038
Diabetes	19(8.56%)	4(5.60%)	15(9.80%)	0.044
Smokers	80(36.04%)	28(40.58%)	52(33.99%)	0.072
Comorbidity, *n* (%)				
Sepsis	19(8.56%)	10(14.49%)	9(5.88%)	<0.001
Pulmonary infection	57(25.68%)	25(36.23%)	32(20.92%)	<0.001
Continuous blood purification	10(4.50%)	4(5.80%)	6(3.92%)	0.185
Mortality, *n* (%)	17(7.66%)	9(13.04%)	8(5.23%)	<0.001
In-bed days(mean ± SD)	35.52 ± 27.45	53.00 ± 31.75	28.32 ± 21.31	<0.001
Length of hospital days(mean ± SD)	72.00 ± 47.54	94.06 ± 52.53	63.05 ± 39.36	<0.001

TBSA, total body surface area; BMI, body mass index; Baux score, age (years) + total body surface area (percent) + (17× inhalation injury).

### Catheter management

Details of catheter management of 630 CVCs with 5,431 line-days are shown in Table 3. CVCs were inserted more frequently on burn wounds in the CLABSI group than the non-CLABSI group (53.39% vs. 39.45%, *p* = 0.006) and were of longer line duration (9.15 ± 4.70 vs. 8.50 ± 5.49, *p* = 0.231), often exceeding 7 days (61.86% vs. 49.61%, *p* = 0.016).

**Table 3.** Catheter management of central venous catheters with and without CLASBI

Feature	Total *n* = 630	CLABSI *n* = 118	Non-CLABSI *n* = 512	*p-*value
Insertion on burn wounds				0.006
Yes	265(42.06%)	63(53.39%)	202(39.45%)	
No	365(57.93%)	55(46.61%)	310(60.55%)	
Anatomical location				0.90
Femoral vein	479(76.03%)	88(74.58%)	391(76.37%)	
Intra jugular vein	108(17.14%)	21(17.80%)	87(17.00%)	
Subclavian vein	43(6.83%)	9(7.63%)	34(6.64%)	
Line-days per CVC	8.62 ± 5.35	9.15 ± 4.70	8.50 ± 5.49	0.231
Line-days				0.016
0–7	303(48.10%)	45(38.14%)	258(50.39%)	
>7	327(51.90%)	73(61.86%)	254(49.61%)	

### Risk factors

Logistic regression analysis was used to screen 17 potential risk factors for CLABSI in burn patients. This showed that longer line-days had the greatest influence on CLABSI (OR = 2.09, *p* = 0.006), followed by insertion on burn wounds (OR = 1.73, *p* = 0.008), more catheterisation times (OR = 1.69, *p* = 0.032), and higher burn index (OR = 1.04, *p* = 0.01) (Table 4).

**Table 4.** Multivariate logistic regression analysis of risk factors for CLABSI in burn patients

Variables	*B*	SE	OR	95% CI	*Wald*	*P*
Longer line-days	0.738	0.269	2.09	1.23–3.55	7.502	0.006
Insertion on burn wounds	0.548	0.207	1.73	1.15–2.60	7.009	0.008
More catheterisation times	0.522	0.244	1.69	1.05–2.72	4.597	0.032
Higher burn index	0.036	0.011	1.04	1.01–1.06	10.409	0.001

### Nomogram predictive model

**Figure 3.**
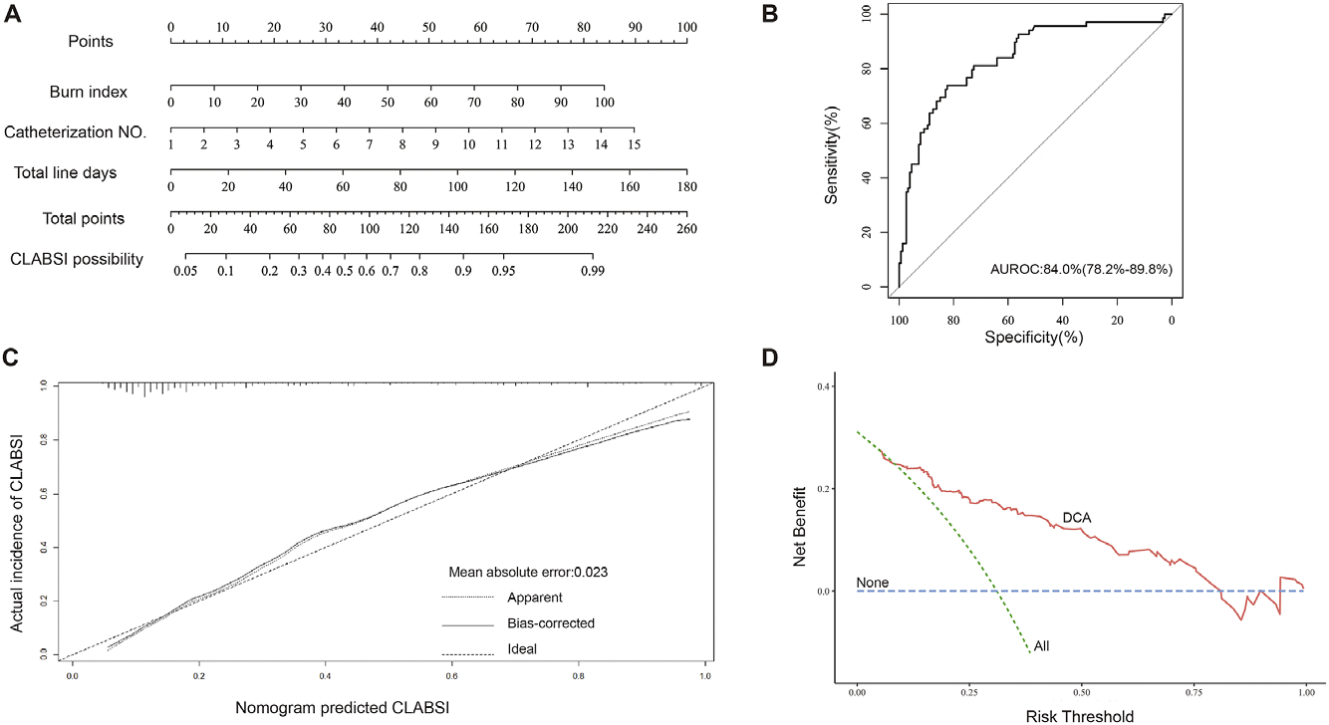
Construction and evaluation of nomogram for predicting CLABSI in burn patients. (a) The developed nomogram for predicting CLABSI in burn patients; (b) ROC curves of nomogram; (c) calibration curve analysis of nomogram; (d) decision curve analysis of nomogram.

Consistent with the foregoing analysis, LASSO regression analysis also found that burn index, catheterisation times, and total line-days were positively related with CLABSI (Figure S1). A nomogram of predicting CLABSI was successfully constructed (Figure 3a) with a maximal AUROC value of 0.84 (95%CI 0.782–0.898) (Figure 3b), and the mean absolute error of the calibration curve was 0.023 (Figure 3c), indicating a good fit. The decision curve analysis indicated that the predictive nomogram provided a good clinical benefit (Figure 3d).

### CLABSI and mortality

Overall, 17 (7.6%) patients died during hospitalisation, with CLABSI patients having a significantly higher mortality rate (13.04% vs. 5.23%, *p* < 0.001). However, Kaplan–Meier survival analysis revealed no significant difference between both groups of patients (*p* = 0.163) (Figure S2). Furthermore, Cox regression analysis showed that the area of full-thickness burns was the only significant risk factor for death (OR = 2.00, *p* = 0.006), and having CLABSI alone was not a significant contributor to mortality (OR = 1.12, *p* = 0.842) (Table S3),

## Discussion

CVCs have become a routine procedure in burn ICU settings, and CLABSI is the most serious and common complication arising from their use [18]. However, confirmation of infection is difficult and resource-costing, mainly relying on local and systemic clinical signs and symptoms and confirmation by catheter and blood cultures [19]. It is necessary for clinicians to show that a bacteremia is due solely to the contamination of the catheter line and exclude all other sources of infection. This requirement may result in delayed diagnosis and clinical intervention. Despite a considerable volume of research defining risk factors for CLABSI, the means to quantitatively predict the infection remain unknown. To address this, we first constructed a practical nomogram for burn patients based on screened risk factors. In addition, we investigated the association of clinical factors with mortality and recorded a number of such factors, namely, the burn index, catheterisation times, total line-days, and CVC insertion on burn wounds. A practical nomogram, based on burn index, catheterisation times, and total line-days, showed excellent predictive ability and clinical applicability.

The incidence of CLABSI in burn patients is significantly higher than that in other populations. In our burn patient cohort, the rate of CLABSI was 21.73 per 1,000 central line-days, which falls within the range reported (15.5–29.1) in other studies in such patients [24-26] – the exception being in Tao’s study [9], which recorded a rate of 29.1 per 1,000 line-days. The main underlying reasons for the high incidence of CLABSI in burn patients are the prolonged demand for central venous access, multiple surgical treatments, and extended stays in the ICU. Other contributory factors are immunosuppression, and breakdown of the skin’s protective barrier. The presence of necrotic tissue and protein-rich exudate at the burn wound surface promotes the multiplication of bacteria on the skin surface at the insertion site which adheres to the outer wall of the catheter before entering the bloodstream. A combination of impaired skin barrier function, haemodynamic changes, and post-traumatic stress render burn patients prone to CRIs. In addition, hypoxia, ischemia, and metabolic abnormalities impact the function of immune cells [20], thereby further increasing the proliferation of opportunistic pathogens.

A high burn area has been identified as a significant risk factor for CRIs [21], but notably, patients in previous studies had significantly less burn area than those found here. Indeed, high burn areas in enrolled patients with CLABSI (68.87% ± 22.31%) appear to have been a contributory factor for its increased incidence in this study. In addition, the CDC defines CLABSI as requiring proof of catheter involvement and other possible sources of infection must be ruled out. In patients with extensive burns, the possibility of infection from the burn wound cannot generally be excluded. All patients who had a positive culture of the catheter tip with the same microbial species found in the blood culture were classified as having a central line–associated infection as opposed to simply being classified as CRI. Therefore, the frequency of CLABSI cases was higher in this study.

Risk factors for CLABSI were complicated and manifold. Our results showed that higher burn index, catheterisation through wounds, puncture times, and longer total line-days were independent risk factors for infection, which was consistent with other studies [21-23]. Apart from burn severity, proper catheter management is widely recognised as the key to preventing infection, but to date, there does not appear to be a consensus on the best strategies of catheter management for infection control. A common controversy is the duration time of central lines. This study found that the incidence of CLABSI was significantly higher in patients with catheterisation for more than 7 days, which is consistent with a recent report [24]. An earlier survey in the USA of national burn units found that 70% of centres had a line duration range of 3–14 days but made no recommendation or guideline for optimal duration regarding the risk of infection [25]. However, another recent study from Australia questioned the practice of regular replacement of deep vein catheters as there was doubt that it reduced the incidence of CLABSI, and possibly even increased the opportunity of infection [26]. Therefore, prospective randomised controlled trials are still needed to clarify the management strategy for CVCs in burn patients.

In this study, the mortality of patients with CLABSI was over two-fold higher than in the non-infected group (13.04% vs. 5.23%). In America, the average reported mortality rate of patients with CLABSI was 12%–25%, and was associated with a 10–20% increase in mortality [27]. Likewise, Hajjej *et al.* documented CLABSI mortality rates as high as 21.8%, compared to 8.3% in controls [28] but found only a weak statistical association between CLABSI and survival time, partly owing to insufficient sample size. Moreover, other studies have shown a strong relationship between CLABSI and mortality [16] and, of note, the finding that ICU-acquired CLABSI was independently associated with higher in-hospital mortality [29].

This study has some limitations. First, the sample size was insufficient to generate a model containing all potential confounding factors. Nevertheless, our sample size was larger than some of the cited studies. Second, this study is intrinsically limited by its retrospective nature, and the level of evidence is possibly inferior to that of a prospective study. Likewise, burn severity and risk of CLABSI were probably higher in this study than in others due to the tertiary status of the burn centre with the referral of patients from other hospitals.

In conclusion, a practical nomogram for predicting CLABSI in burn patients showed good predictive ability and clinical applicability. Key risk factors of the nomogram included burn index, catheterisation times, and total line-days. Further studies are warranted to improve the efficacy and applicability of the nomogram in other settings.

## Data Availability

All data generated and/or analysed are available from the corresponding author on reasonable request.

## Abbreviations

CLABSI: central line–associated bloodstream infection
CRI: catheter-related infection
CVCs: central venous catheters

## Supplementary material

For supplementary material accompanying this paper visit http://doi.org/10.1017/S0950268823000766. click here to view supplementary material

## References

[1] Logsetty S, Shamlou A, Gawaziuk JP, March J, Doupe M, Chateau D, Hoppensack M, Khan S, Medved M, Leslie WD, Enns MW, Stein MB, Asmundson GJG and Sareen J (2016) Mental health outcomes of burn: A longitudinal population-based study of adults hospitalized for burns. Burns 42(4), 738–744. 27049068 10.1016/j.burns.2016.03.006

[2] Rybarczyk MM, Schafer JM, Elm CM, Sarvepalli S, Vaswani PA, Balhara KS, Carlson LC, Jacquet GA (2017) A systematic review of burn injuries in low- and middle-income countries: Epidemiology in the WHO-defined African Region. African Journal of Emergency Medicine 7(1), 30–37. 30456103 10.1016/j.afjem.2017.01.006 PMC6234151

[3] Li H, Yao Z, Tan J, Zhou J, Li Y, Wu J and Luo G (2017) Epidemiology and outcome analysis of 6325 burn patients: A five-year retrospective study in a major burn center in Southwest China. Scientific Reports 7, 46066. 28383066 10.1038/srep46066 PMC5382583

[4] Govindan S, Snyder A, Flanders SA and Chopra V (2018) Peripherally inserted central catheters in the ICU: A retrospective study of adult medical patients in 52 hospitals. Critical Care Medicine 46(12), e1136–e1144. 30247241 10.1097/CCM.0000000000003423 PMC6317857

[5] Gotchac J, Poullenot F, Guimber D, Ecochard-Dugelay E, Schneider S, Peretti N, Billiauws L, Borderon C, Breton A, Chaillou Legault E, Chambrier C, Comte A, Coste ME, Djeddi D, Dubern B, Dupont C, Espeso L, Fayemendy P, Flori N, Fotsing G, Gastineau S, Goulet O, Guiot E, Jirka A, Languepin J, Layec S, Quilliot D, Rebouissoux L, Seguy D, Talon I, Turquet A, Vallee M, Willot S, Lamireau T and Enaud R (2022) Management of Central Venous Catheters in children and adults on home parenteral nutrition: A French survey of current practice. Nutrients 14(12), 2532. 35745262 10.3390/nu14122532 PMC9227599

[6] Allon M, Brouwer-Maier DJ, Abreo K, Baskin KM, Bregel K, Chand DH, Easom AM, Mermel L, Mokrzycki MH, Patel PR, Roy-Chaudhury P, Shenoy S, Valentini RP and Wasse H (2018) Recommended clinical trial end points for dialysis catheters. Clinical Journal of the American Society of Nephrology 13(3), 495–500. 28729382 10.2215/CJN.12011116 PMC5967684

[7] Li C, Wang S, Wang H, Wu Y, Ma J, Li W and Duan J (2021) The effects of hemodynamic monitoring using the PiCCO system on critically ill patients. American Journal of Translational Research 13(9), 10578–10585. 34650729 PMC8507053

[8] Karagiannidou S, Zaoutis T., Maniadakis N, Papaevangelou V and Kourlaba G (2019) Attributable length of stay and cost for pediatric and neonatal central line-associated bloodstream infections in Greece. Journal of Infection and Public Health 12(3), 372–379. 30616938 10.1016/j.jiph.2018.12.004

[9] Chovanec K, Arsene C, Gomez C, Brixey M, Tolles D, Galliers JW, Kopaniasz R, Bobash T and Goodwin L (2021) Association of CLABSI with hospital length of stay, readmission rates, and mortality: A retrospective review. Worldviews on Evidence-Based Nursing 18(6), 332–338. 34779128 10.1111/wvn.12548

[10] Rosenthal VD, Jin Z, Memish ZA, Daboor MA, al- Ruzzieh MA, Hussien NH, Guclu E, Olmez-Gazioglu E, Ogutlu A, Agha HM, el-Sisi A, Fathalla AA, Yildizdas D, Yildizdas HY, Ozlu F, Horoz OO, Omar AA, Belkebir S, Kanaa A, Jeetawi R, el-Kholy AA, Bayani V, Alwakil W, Abdulaziz-Alkhawaja S, Swar SF, Magray TA, Alsayegh AA and Yin R (2022) Risk factors for mortality in ICU patients in 10 middle eastern countries: The role of healthcare-associated infections. Journal of Critical Care 72, 154149. 36108349 10.1016/j.jcrc.2022.154149

[11] Gominet M, Compain F, Beloin C and Lebeaux D (2017) Central venous catheters and biofilms: Where do we stand in 2017? APMIS 125(4), 365–375. 28407421 10.1111/apm.12665

[12] Ziegler MJ, Pellegrini DC and Safdar N (2015) Attributable mortality of central line associated bloodstream infection: Systematic review and meta-analysis. Infection 43(1), 29–36. 25331552 10.1007/s15010-014-0689-y

[13] Garcia RA, Spitzer ED, Beaudry J, Beck C, Diblasi R, Gilleeny-Blabac M, Haugaard C, Heuschneider S, Kranz BP, McLean K, Morales KL, Owens S, Paciella ME and Torregrosa E (2015) Multidisciplinary team review of best practices for collection and?Handling of blood cultures to determine effective interventions for?Increasing the yield of true-positive bacteremias, reducing contamination, and eliminating false-positive central line-associated bloodstream infections. American Journal of Infection Control 43(11), 1222–1237. 26298636 10.1016/j.ajic.2015.06.030

[14] Wang X, Lu J, Song Z, Zhou Y, Liu T and Zhang D (2022) From past to future: Bibliometric analysis of global research productivity on nomogram (2000-2021). Frontiers in Public Health 10, 997713. 36203677 10.3389/fpubh.2022.997713 PMC9530946

[15] Zhang Z, Brusasco C, Anile A, Corradi F, Mariyaselvam M, Young P, Almog Y, du B, Yu X, Zhu H, Zhang M, Cao Y and Hong Y (2018) Clinical practice guidelines for the management of central venous catheter for critically ill patients. Journal of Emergency and Critical Care Medicine 2(5), 53.

[16] Magiorakos AP, Srinivasan A, Carey RB, Carmeli Y, Falagas ME, Giske CG, Harbarth S, Hindler JF, Kahlmeter G, Olsson-Liljequist B, Paterson DL, Rice LB, Stelling J, Struelens MJ, Vatopoulos A, Weber JT and Monnet DL (2012) Multidrug-resistant, extensively drug-resistant and pandrug-resistant bacteria: An international expert proposal for interim standard definitions for acquired resistance. Clinical Microbiology and Infection 18(3), 268–281. 21793988 10.1111/j.1469-0691.2011.03570.x

[17] NHSN. *Patient Safety Component Manual, Chapter 4, Bloodstream Infection Event (Central Line-Associated Bloodstream Infection and Non-central Line Associated Bloodstream Infection) https://wwwcdcgov/nhsn/pdfs/pscmanual/pcsmanual_currentpdf2021*, 2021.

[18] Zeng C, Wu A, Li L and Jia H (2021) Multi-center prospective study on central line-associated bloodstream infections in 79 ICUs of China. BMC Infectious Diseases 21(1), 1208. 34863108 10.1186/s12879-021-06871-5 PMC8642979

[19] Aldea MC, et al. (2019) Microbiological diagnosis of catheter-related infections.Enfermedades Infecciosas Y. Microbiologia Clinica 37(10), 668–672. 10.1016/j.eimc.2018.07.009 30220518

[20] Mulder P, Koenen HJPM, Vlig M, Joosten I, de Vries RBM and Boekema BKHL (2022) Burn-induced local and systemic immune response: Systematic review and meta-analysis of animal studies. Journal of Investigative Dermatology 142(11), 3093–3109. 35623415 10.1016/j.jid.2022.05.004

[21] Fochtmann-Frana A, et al. (2018) Incidence of risk factors for bloodstream infections in patients with major burns receiving intensive care: A retrospective single-center cohort study. Burns 44(4), 784–792. 29395408 10.1016/j.burns.2017.12.009

[22] Castelli GP, Pognani C, Stuani A, Cita M and Paladini R (2007) Central venous catheter replacement in the ICU: New site versus guidewire exchange. Minerva Anestesiologica 73(5), 267–273. 17159763

[23] Friedman BC, Mian MAH, Mullins RF, Hassan Z, Shaver JR and Johnston KK (2015) Five-lumen antibiotic-impregnated femoral central venous catheters in severely burned patients: An investigation of device utility and catheter-related bloodstream infection rates. Journal of Burn Care & Research 36(4), 493–499. 25407386 10.1097/BCR.0000000000000186

[24] Rickard CM, Marsh NM, Larsen EN, McGrail MR, Graves N, Runnegar N, Webster J, Corley A, McMillan D, Gowardman JR, Long DA, Fraser JF, Gill FJ, Young J, Murgo M, Alexandrou E, Choudhury MA, Chan RJ, Gavin NC, Daud A, Palermo A, Regli A and Playford EG (2021) Effect of infusion set replacement intervals on catheter-related bloodstream infections (RSVP): a randomised, controlled, equivalence (central venous access device)-non-inferiority (peripheral arterial catheter) trial. Lancet (London, England) 397(10283), 1447–1458. 33865494 10.1016/S0140-6736(21)00351-2

[25] Sheridan RL, Neely AN, Castillo MA, Shankowsky HA, Fagan SP, Chung KK and Weber JM (2012) A survey of invasive catheter practices in U.S. burn centers. Journal of Burn Care & Research 33(6), 741–746. 23147213 10.1097/BCR.0b013e318254d4ab

[26] Pearse I, Corley A, Rickard CM and Marsh N (2021) Unnecessary removal of vascular access devices due to suspected infection in Australian intensive care units. Australian Critical Care 35(6), 644–650. 34711493 10.1016/j.aucc.2021.09.005

[27] Liang SY and Marschall J (2011) Update on emerging infections: News from the Centers for Disease Control and Prevention. Vital signs: Central line-associated blood stream infections--United States, 2001, 2008, and 2009. Annals of Emergency Medicine 58(5), 447–451. 22018400 10.1016/j.annemergmed.2011.07.035

[28] Hajjej Z, Nasri M, Sellami W, Gharsallah H, Labben I, Ferjani M. (2014) Incidence, risk factors and microbiology of central vascular catheter-related bloodstream infection in an intensive care unit. Journal of Infection and Chemotherapy 20(3), 163–168. 24508422 10.1016/j.jiac.2013.08.001

[29] Wong SW, Gantner D, McGloughlin S, Leong T, Worth LJ, Klintworth G, Scheinkestel C, Pilcher D, Cheng AC and Udy AA (2016) The influence of intensive care unit-acquired central line-associated bloodstream infection on in-hospital mortality: A single-center risk-adjusted analysis. American Journal of Infection Control 44(5), 587–592. 26874406 10.1016/j.ajic.2015.12.008

