# Supplementary material for: Clinical epidemiology and a novel predicting nomogram of central line associated bloodstream infection in burn patients
Source: Epidemiol Infect. 2023 May 23;151:e90. doi: 10.1017/S0950268823000766 (PMC10265734; doi:10.1017/S0950268823000766)
Supplement: Supplementary file 1 [file S0950268823000766sup001.doc]

**
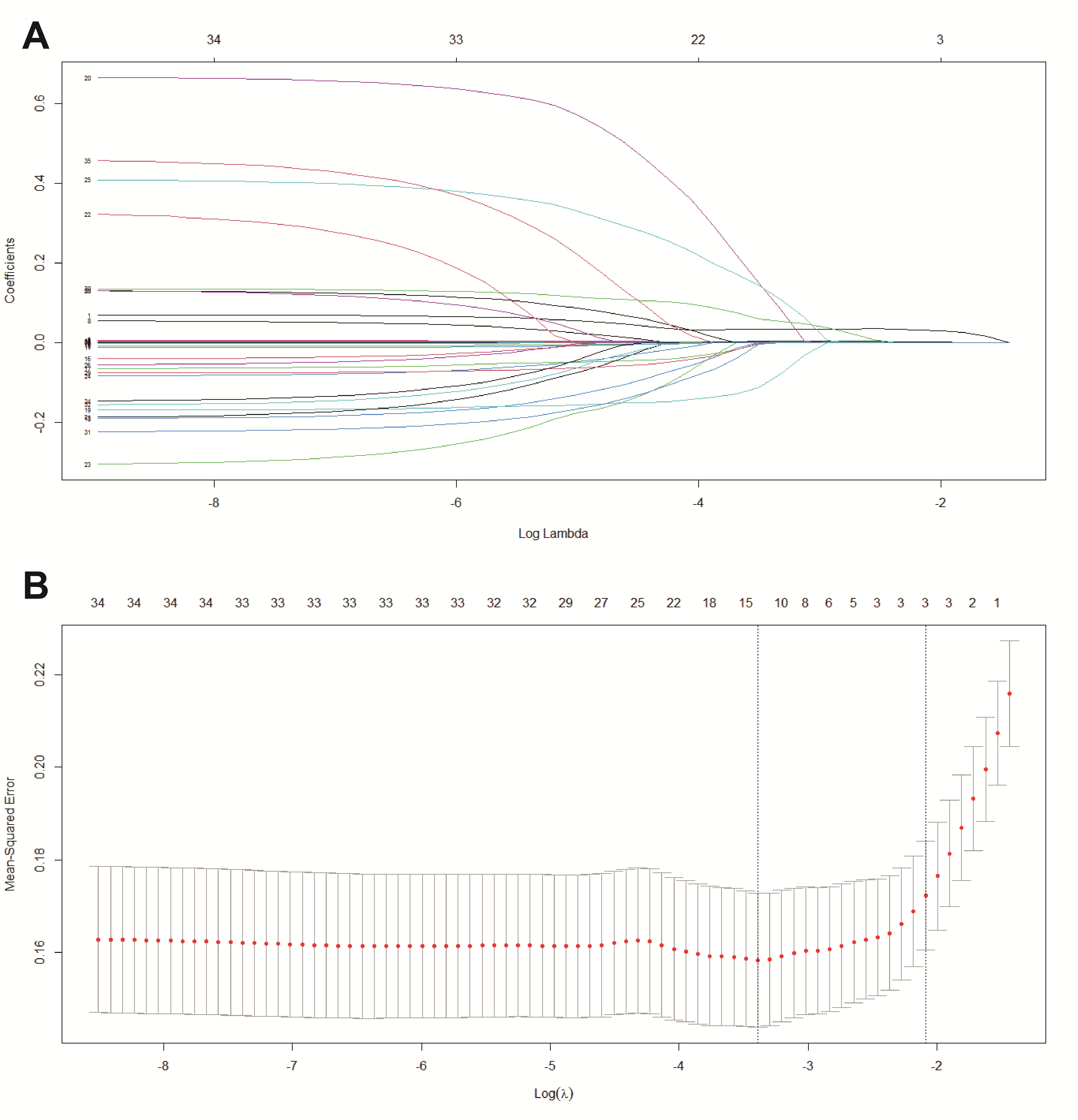
**

**Figure S1. LASSO regression analysis of 17 variables.**

A. Features’ selection using LASSO regularization. LASSO coefficient profiles (y-axis) of the 17 features. The lower x-axis indicates the log (λ). The top x-axis indicates the average number of predictors. B. Identification of the optimal penalization coefficient (λ) in the LASSO model based on mean squared error. LASSO, Least-absolute shrinkage and selection operator.

**
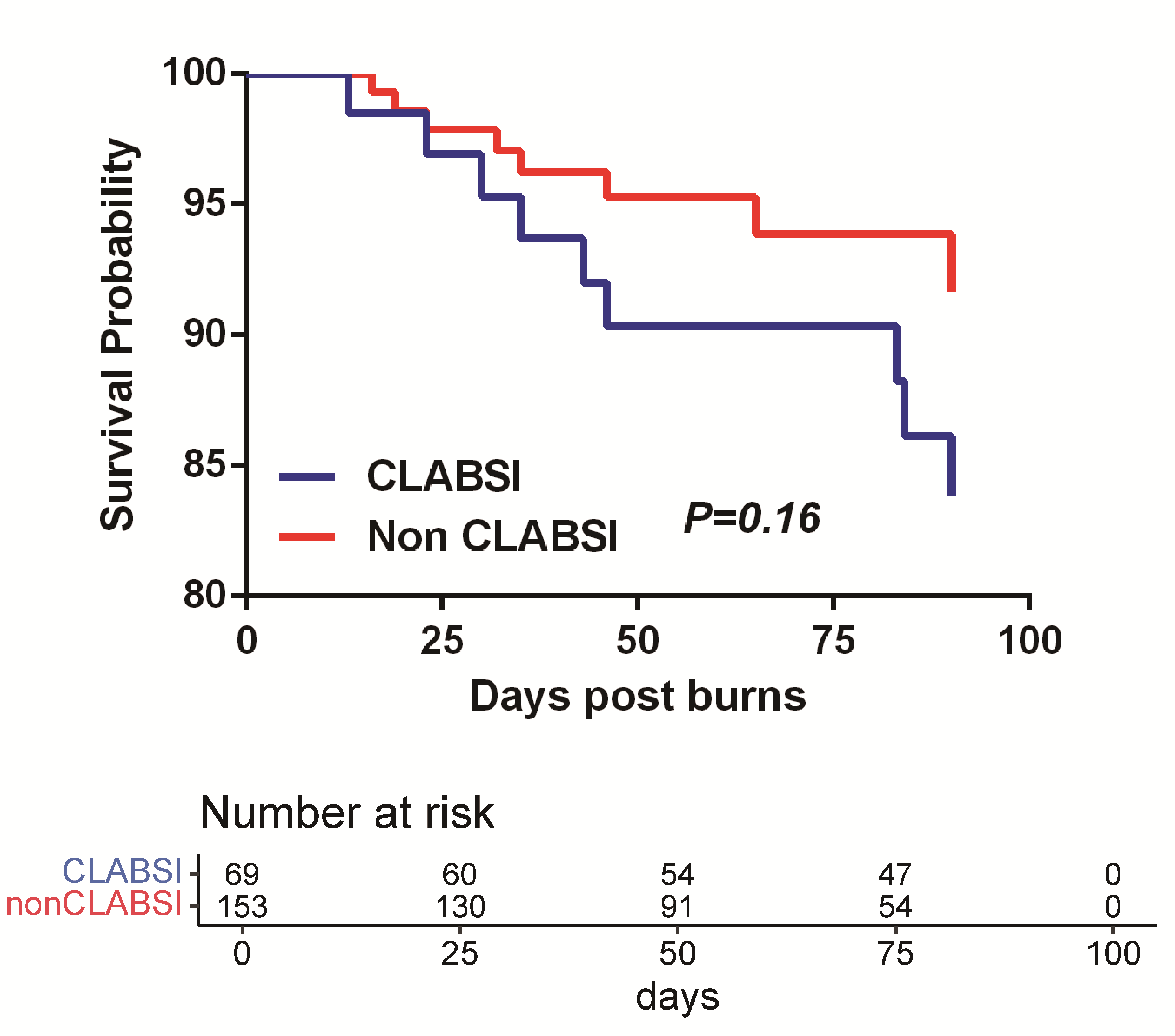
**

**Figure S2. Kaplan–Meier survival analysis of burn patients with or without CLABSI**

**Table S1. Assignment of the collected 17 factors in the Logistic regression analysis of CLABSI**

| **Risk factors** | **Variables** | **Evaluation** |
| --- | --- | --- |
| Gender | X1 | Male=1, Female=0 |
| Age | X2 | Quantitative value |
| BMI | X3 | Quantitative value |
| Burn cause | X4 | Flame&scald=1, Others=0 |
| Baux score | X5 | Quantitative value |
| Burn index | X6 | Quantitative value |
| Full-thickness burns | X7 | Quantitative value |
| Hyperlipidemia | X8 | Yes=1, No=0 |
| Hormone therapy | X9 | Yes=1, No=0 |
| Diabetes | X10 | Yes=1, No=0 |
| Smoking | X11 | Yes=1, No=0 |
| Hypertension | X12 | Yes=1, No=0 |
| Inhalation injury | X13 | Yes=1, No=0 |
| Days post injury | X14 | 0-7d=1,8-14d=2,15-21d=3,>21d=4 |
| Line days | X15 | 0-7d=1,8-14d=2,15-21d=3,>21d=4 |
| Catheterization on wound | X16 | Yes=1, No=0 |
| Catheterization times | X17 | 0=0,1=1,2=2,3=3,4 or more=4 |
| CLABSI | Y | Yes=1, No=0 |

**Table S2. The pathogen distribution of CLABSI in burn patients**

| **Strains** | **Number** | **Percentage (%)** |
| --- | --- | --- |
| **Gram-negative bacteria** | **124** | **76.07%** |
| *Acinetobacter baumannii* | 64 | 39.26% |
| *Pseudomonas aeruginosa* | 30 | 18.40% |
| *Stenotrophomonas maltophilia* | 13 | 7.98% |
| *Klebsiella pneumoniae* | 8 | 4.91% |
| *Serratia marcescens* | 3 | 1.84% |
| *Enterobacter cloacae* | 2 | 1.23% |
| *colon bacillus* | 2 | 1.23% |
| *Others* | 2 | 1.23% |
| **Gram-positive bacteria** | **39** | **23.93%** |
| *Staphylococcus aureus* | 21 | 12.88% |
| *Enterococcus faecium* | 6 | 3.68% |
| *Staphylococcus haemolyticus* | 5 | 3.07% |
| *Staphylococcus epidermidis* | 4 | 2.45% |
| *Others* | 3 | 1.84% |

**Table S3.** **Risk factors of mortality by COX regression analysis**

| **Variables** | **B** | **SE** | ***Wald*** | **OR** | **95% CI** | | ***P*** | |
| --- | --- | --- | --- | --- | --- | --- | --- | --- |
| **With Inhalation injury** | 1.477 | 0.778 | 3.605 | 4.380 | 0.95-20.12 | 0.058 | |  |
| **Area of full-thickness burns** | 0.694 | 0.250 | 7.696 | 2.003 | 1.22-3.27 | 0.006 | |  |
| **With CLABSI** | 0.109 | 0.547 | 0.040 | 1.115 | 0.38-3.26 | 0.842 | |  |
| **Older age** | 0.013 | 0.016 | 0.658 | 1.013 | 0.98-1.05 | 0.417 | |  |
| **Higher BMI** | -0.017 | 0.059 | 0.081 | 0.983 | 0.88-1.10 | 0.775 | |  |
| **Male** | -0.938 | 0.573 | 2.683 | .391 | 0.13-1.20 | 0.101 | |  |
